# Effects of HIIT at different frequencies in an aquatic environment on mental health in multimorbid older people: A randomized clinical trial

**DOI:** 10.1016/j.clinsp.2025.100803

**Published:** 2025-10-11

**Authors:** Vitória Oliveira Silva da Silva, Anand Thirupathi, Rafael Alex dos Santos Macedo, Mauricio Fagundes Santos, Ana Claudia de Oliveira Borba, Yaodong Gu, Karin Martins Gomes, Rodrigo Sudatti Delevatti, Paulo Cesar Lock Silveira, Luciano Acordi da Silva

**Affiliations:** a Research Academy of Medicine Combining Sports, Ningbo No 2 Hospital, Ningbo, China; b Post-grad Programme in Health Sciences, Universidade do Extremo Sul Catarinense, Santa Catarina, SC, Brazil; c Undergraduate Programme, Psychology, Universidade do Extremo Sul Catarinense, Santa Catarina, SC, Brazil; d Undergraduate Programme, Physical Education, Universidade do Extremo Sul Catarinense, Santa Catarina, SC, Brazil; e Post-grad Programme in Physical Education, Universidade Federal de Santa Catarina, Santa Catarina, SC, Brazil

**Keywords:** Physical exercise, Mental health, Training frequency, Older People, Multimorbidity

## Abstract

**Background:**

This study aimed to compare the chronic effects of High-Intensity Interval Training (HIIT) aqua aerobics performed once a week (G1) versus twice a week (G2) on sleep quality, daytime sleepiness, anxiety, and depression in older adults with multimorbidities.

**Methods:**

Both groups performed the same exercises (9 in total) with identical volume (4 sets of 30 seconds of activity followed by 30 seconds of rest) and intensity (80 %–90 % of Heart Rate max, corresponding to 8–9 on the Borg Scale), in 40-minute sessions. Mental health parameters were assessed 48 hours after the last training session. To analyze the effects of HIIT, a two-way analysis of variance (anova), with Bonferroni post hoc tests, was used with a significance level set at *p* < 0.05.

**Results:**

Initially, one hundred and twenty-nine older adults were assessed for eligibility. Fifty-six older adults with multimorbidities, including diabetes, hypertension, and depression, completed the study. Significant improvements were observed only in G2 (sessions performed twice a week), with increases in sleep quality (+49 %, *p* = 0.032) and reductions in daytime sleepiness (-50 %, *p* = 0.041), anxiety (-45 %, *p* = 0.047), and depression (-62 %, *p* = 0.017).

**Conclusions:**

The results suggest that HIIT aqua aerobics improves mental health outcomes in older adults with multimorbidities only when performed at least twice a week.

**Trial registration:**

Brazilian Registry of Clinical Trials (ReBEC) RBR-9kqwd5f. Registered on January 14, 2024.

## Highlights

• HIIT aqua aerobics twice a week improves sleep, anxiety, and depression in older adults.
• Mental health benefits were observed only with a training frequency of twice per week.
• HIIT in water was safe and well-tolerated by multimorbid elderly participants.

## Introduction

Multimorbidity (defined as the presence of two or more chronic diseases in the same individual) is strongly associated with aging.^1^ The prevalence of multimorbidity in older Brazilian people is 53.1 %, and studies indicate that this number is expected to increase further.^2^ Among the most common conditions affecting older people with multimorbidity are diabetes, hypertension, and depression.

Older individuals with multimorbidity often experience mental health disorders, such as depression and anxiety, which exacerbate the progression of their chronic conditions, creating a vicious cycle.^3^ Addressing mental health is crucial, as it can reduce stress levels, strengthen emotional resilience, and improve adherence to treatment plans. Crucially, mental health also profoundly impacts sleep quality, and conversely, good sleep is fundamental not only for mental well-being but also for overall physiological restoration and cognitive function, influencing daily performance beyond psychological aspects.^4^ These factors collectively contribute to better control of multimorbidity by mitigating both its physical and psychological impacts.

In addition to the direct impact on the progression of chronic diseases, the deterioration of mental health in older adults with multimorbidity has been consistently associated with a significant reduction in functionality.^5^ Conditions such as depression and anxiety can lead to decreased motivation for daily activities, impaired mobility, social isolation, and less engagement in health behaviors, such as adherence to treatments and physical exercise.^6^ This decrease in functionality not only compromises the quality of life of the elderly but also increases dependence on caregivers and health costs, creating a vicious cycle that aggravates multimorbidity and its negative outcomes.^7^ Therefore, interventions aimed at improving mental health are crucial to preserving and optimizing functionality in this population.

Aquatic physical training, on the other hand, has long been recognized as an effective strategy to prevent and manage various diseases,^8^, ^9^, ^10^ particularly in older people with multimorbidity, due to the specific properties of water, such as buoyancy, hydrostatic pressure, and thermal conductivity.^11^ According to Mazetti,^12^ these characteristics reduce joint overload, promote muscle relaxation, and enhance circulation, contributing to improved physical function and psychological well-being. Additionally, the controlled environment of aquatic exercise offers a unique context for stress reduction, further supporting mental health in this population.

Studies have demonstrated significant improvements in mental health in older people with depression, diabetes, and hypertension after regular low- and moderate-intensity physical training in an aquatic environment.^6^^,^^13^, ^14^, ^15^ However, there is a gap in the literature regarding the effects of high-intensity training on older people with multimorbidity.

Notably, the positive effects reported in the literature are often associated with training frequencies of at least two sessions per week, totaling at least 75 minutes per week of vigorous-intensity exercise or 150 minutes of moderate-intensity activity.^16^ However, adherence and long-term commitment to exercise programs remain challenges, with lack of time being a frequently cited barrier. Given this, investigating the effects of minimal doses of aquatic exercise, particularly through low-frequency (1 × and 2 × per week) and time-efficient protocols like High-Intensity Interval Training (HIIT), is essential. In this study, the weekly training volume in the 1 × and 2 × per week conditions will be 40 and 80 minutes per week, respectively, considerably lower than the typical recommendations.

Therefore, this study aims to compare the chronic effects of water aerobics High-Intensity Interval Training (HIIT) (1 × per week vs. 2 × per week) in an aquatic environment on sleep, sleepiness, anxiety, and depression in older people with multimorbidity. The authors hypothesize that two weekly physical training sessions in an aquatic environment are necessary to significantly improve the mental health of older people with multimorbidity.

## Methods

### Study design

This research is based on a randomized clinical trial with a comparative design. Participants were randomly allocated to two groups: one received the intervention once a week (G1), while the other received the intervention twice a week (G2). The allocation was performed through simple randomization via an online draw, with each participant assigned a unique number. The study is registered in the Brazilian Registry of Clinical Trials, number RBR-9kqwd5f. The study was also approved by a Research Ethics Committee and conducted in accordance with the principles of the Declaration of Helsinki. This study complies with all CONSORT guidelines and reports the required information accordingly.

### Participants

Older people were recruited from the integrated clinics of the university in Criciúma, Brazil. These individuals were identified based on a list of those diagnosed with multimorbidity, including depression, diabetes, and hypertension. Potential participants were contacted by phone and invited to join the study. All participants had medical clearance for physical exercise, provided through medical certificates. Participants were divided into two groups: Group 1 (aqua aerobics once a week) and Group 2 (aqua aerobics twice a week).

Inclusion criteria were: 1) Age > 60-years; 2) Medical certificate confirming at least two of the three conditions (depression, diabetes, and hypertension); 3) Medical certificate authorizing underwater physical exercise; 4) Proof of three COVID-19 vaccination doses. Exclusion criteria were: 1) Clinical contraindications to exercise; 2) Diagnosis of other conditions that could interfere with study results; 3) Missing required documents; 4) Age < 60-years.

All participants were informed about the study’s objectives, procedures, potential risks, and benefits before providing written informed consent. The study adhered to the ethical principles outlined in Resolution 466/12 of the National Health Council (CNS). Confidentiality and anonymity of the data were maintained throughout the study.

### Randomized and blinded

The participants were allocated to the groups by simple randomization, using an internet lottery. Each older person was assigned a unique number, and the participants were randomly assigned to the groups. The allocation sequence was generated by a researcher who was not directly involved in the study. The allocation list was kept hidden from the outcome assessors throughout the study and was kept confidential from the participants and aquatic exercise instructors until the treatments were assigned.

Due to the nature of the intervention (same modality of aqua aerobics), with a difference only in frequency (once a week or twice a week), the participants were aware of the frequency of the intervention they were undertaking, and the instructors were also aware of the participants’ allocation, which made it impossible to blind the participants and instructors. However, all outcome assessors remained blind to the participant group and the study hypothesis, minimizing assessment bias.

### Interventions

The sessions were conducted by two research group scholarship holders under the supervision of professors. One scholarship holder remained outside the pool, while the other was in the water, assisting with guidance and exercise monitoring. The pool used had a length of 25 meters, a thermoneutral temperature (approximately 28‒30 °C), and a depth of 1.20 meters.

Participants underwent aquatic training once or twice a week, depending on group allocation, for a total duration of 12 weeks. Each session lasted 40 minutes, structured as follows: 5 minutes of warm-up, 30 minutes of main training, and 5 minutes of cooldown. The main training consisted of eight exercises targeting the core, upper limbs, and lower limbs, performed using an interval method (Table 1).

**Table 1:** Hydro-HIIT protocol.

Exercise	Series	Work time	Interval series	Interval exercise	Intensity
Hydro-HIIT	4	*30 s*	*30* sec	*60 s*	8‒10 (CR10)
Total: 8 exercises, 32 series, 40 min of Hydro-HIIT.					

Group 1 (1 × /week): Participants performed hydro-gymnastics once a week following the described session structure. The main training was divided into four sets of 30 seconds of exercise, interspersed with 30 seconds of active recovery (light stationary running) and 1-minute rest between exercises.

Group 2 (2 × /week): Participants followed the same training protocol as Group 1, with the only difference being two weekly sessions (Table 1).

To ensure proper training intensity control, heart rate monitors were used, with target intensities of 80 %–90 % of Heart Rate max during active phases (exercise) and < 60 % of Heart Rate max during passive phases (active rest and stationary running). Given the influence of water immersion on heart rate response, Heart Rate max was adjusted using individual correction factors, following the equation proposed by Graef and Kruel (36): HeartRatemaxwater=HeartRatemaxland−ΔHeartRate. Additionally, the Borg Rating of Perceived Exertion scale (0–10) was used, with values between 8 and 10 (“very intense” and “maximum”) for high-intensity exercise moments and 1 to 3 (“very light” and “extremely light”) for recovery periods.^17^

Heart rate was continuously monitored during the sessions to ensure participants maintained the target intensity during the active phases. The exercise prescription, based on High-Intensity Interval Training (HIIT), alternated between periods of intense exercise and short active rest phases, aiming for cardiovascular benefits while considering the participants’ health conditions. The protocol was tailored to individual needs, ensuring both safety and effectiveness.

The high-intensity interval training protocol was developed to use the intrinsic resistance of water (drag) as the main form of overload. This resistance is based on the Theoretical Square Law, which states that the drag force imposed by the aquatic environment increases proportionally to the square of the speed of movement. To ensure adequate intensity, the exercises were designed to be performed at maximum speed and range of motion, maximizing the effort against water drag.^18^

It is important to note that no dumbbells, floats, or any other additional equipment were used to modify or increase resistance. The overload was determined exclusively by the interaction of the participant's body with the water and the intensity of the movements performed.

### Measurements

Before starting and 48 hours after completing the exercise programs, mental health assessments were conducted by psychologists, using a blinded approach for all tests.

Sleep quality was assessed using the *Pittsburgh Sleep Quality Index* (PSQI),^19^ which classifies participants as “good” or “poor” sleepers based on their responses. This instrument evaluates overall sleep quality, ranging from 0 to 21, where higher scores indicate poorer sleep quality. A global PSQI score > 5 indicates poor sleep quality (significant sleep difficulties), while scores ≤ 5 indicate good sleep quality.

Daytime sleepiness was measured with the *Epworth Sleepiness Scale* (ESS),^20^ where participants rated their likelihood of dozing off in eight different situations. This self-administered scale has a global score ranging from 0 to 24. A global ESS score > 10 suggests the diagnosis of Excessive Daytime Sleepiness (EDS).

Anxiety was evaluated using the Beck Anxiety Inventory (BAI),^21^ a 21-item scale that assesses symptoms of anxiety experienced over the past week. Scores are categorized as follows: 0‒7 (minimal), 8‒15 (mild), 16‒25 (moderate), and 26‒63 (severe).

Depression was measured using the Beck Depression Inventory (BDI),^21^ which includes 21-items covering cognitive, affective, behavioral, and somatic aspects of depression. Depression severity was categorized based on the total score. The BDI scores indicate depression severity as follows: 0‒13 (minimal), 14‒19 (mild), 20‒28 (moderate), and ≥ 29 (severe).

### Sample size calculation

It was performed by analysing the mean comparison between the groups, setting a significance level or alpha at 5 % (type I error), with a sample power of 80 % (beta or type II error at 20 %). The sample size was estimated according to mean values and standard deviation from previous studies conducted by the present group, with diabetic and depressed subjects undergoing aquatic exercises for mental health score analysis.^6^^,^^13^

### Statistical analysis

All information collected from questionnaires and tests was tabulated in an Excel spreadsheet. Statistical analyses were performed using a two-way analysis of variance (ANOVA), followed by the post hoc Bonferroni test. Data normality was verified using the Shapiro-Wilk test. The effect size was calculated by Cohen's test, and the comparison between groups was conducted using the Eta-square test. The significance level established for the statistical test was *p* < 0.05. Statistical Package for the Social Sciences (SPSS) version 22.0 was used as a statistical package. Data were compared with previously established standards and qualitatively classified when necessary.

For the primary mental health outcomes, participants who dropped out of the study (2 in G1 and 2 in G2) or who had inadequate adherence (< 90 % attendance in classes) (1 in G1 and 2 in G2) were thus excluded from the final analyses. Consequently, the final sample analyzed consisted of 21 participants in G1 and 35 in G2. This approach, characterized as a per-protocol analysis, allowed us to evaluate the effects of training in individuals who completed the protocol as planned, which was crucial for determining the efficacy of the proposed intervention frequency. Missing data resulting from these exclusions were not imputed.

## Results

A total of 129 participants were selected for inclusion in the study. Of these, 73 were excluded, mainly for not meeting the inclusion criteria (*n* = 52) or declining to participate (*n* = 21). The remaining 63 participants were allocated into one of two groups: water aerobics once a week (G1, *n* = 24) or water aerobics twice a week (G2, *n* = 39).

**Fig. 1:**
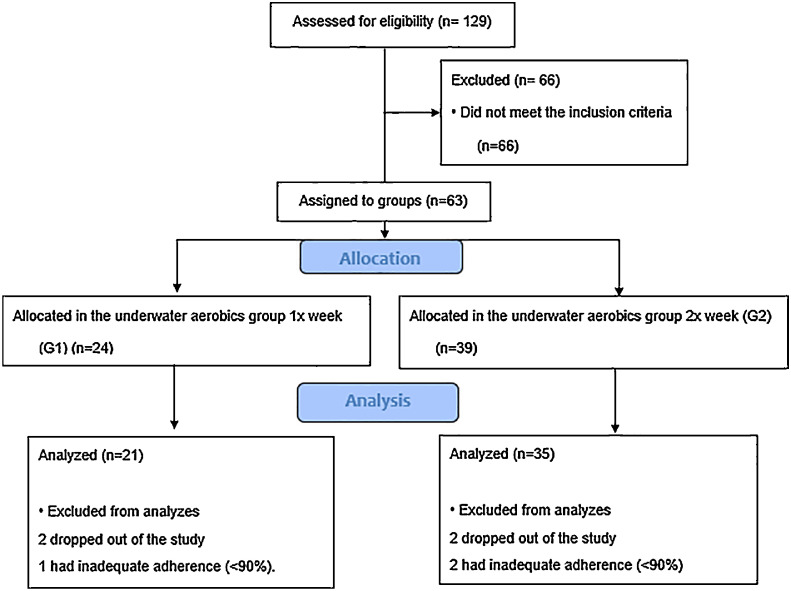
Diagram of the sample selection process.

During the intervention, some participants dropped out or had inadequate adherence (< 90 % attendance in classes) and were thus excluded from the analyses, leading to a final sample of 21 participants in G1 and 35 in G2 for analysis. The participant flowchart is illustrated in Fig. 1.

A total of 56 older people with multimorbidity were evaluated, consisting of most females (50 women and 6 men) aged between 62 and 77 years (70.81 ± 5.54; 67.26 ± 4.69). Both G1 and G2 presented Body Mass Index (BMI) > 30, characterizing Grade 1 obesity.

### Characterization of high-intensity interval training (HIIT)

**Fig. 2:**
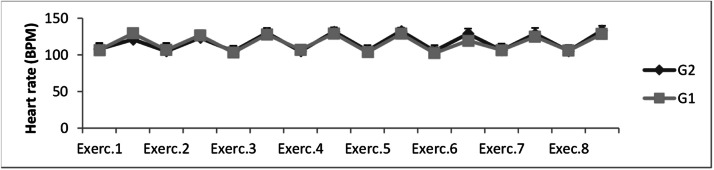
Evaluation of heart rate (BPM) of the sample of this study. Caption: BPM, Heart rate assessment. Note: Results refer to heart rate levels in G1 and G2 before and after exercise. G1, Group that practiced water aerobics 1 × *a* week; G2, Group that practiced water aerobics 2 × *a* week. The reported values are the averages of the first, sixth, and twelfth week of training. The symbol (*) indicates intragroup statistical differentiation. The significance level was *p* < 0.05.

The intensity of the HIIT, measured in Heart Rate (beats per minute), can be observed in Fig. 2. The Heart Rate behavior during exercises significantly varied, characterizing the interval method (*p* < 0.05). On G1 group, values increased significantly in Exercise 1 (107 ± 8.8 before; 129 ± 6.2 at the end); Exercise 2 (107 ± 9.2 before; 127 ± 7.2 at the end); Exercise 3 (103 ± 8.8 before; 128 ± 7.9 at the end); Exercise 4 (107 ± 4.8 before; 129 ± 7.2 at the end); Exercise 5 (104 ± 5.5 before; 129 ± 6.6 at the end); Exercise 6 (102 ± 8.3 before; 119 ± 5.6 at the end); Exercise 7 (107 ± 8.5 before; 125±7.3 at the end); and Exercise 8 (106 ± 7.9 before; 129 ± 7.3 at the end). On G2 group, values also increased significantly in Exercise 1 (108 ± 8.3 before; 121 ± 7.7 at the end); Exercise 2 (105 ± 10.1 before; 123 ± 5.9 at the end); Exercise 3 (105 ± 7.5 before; 130 ± 6.9 at the end); Exercise 4 (105 ± 5.1 before; 132 ± 5.9 at the end); Exercise 5 (106 ± 7.5 before; 133 ± 4.6 at the end); Exercise 6 (105 ± 7.9 before; 129 ± 6.9 at the end); Exercise 7 (106 ± 9.2 before; 129 ± 8.2 at the end); and Exercise 8 (105 ± 4.6 before; 133 ± 6.1 at the end). The range of average heart rate and subjective perception of effort before and at the end of each exercise represents, for older people, an effort between 80 % and 90 % of Heart Rate max and a very intense effort characterizing HIIT.

**Fig. 3:**
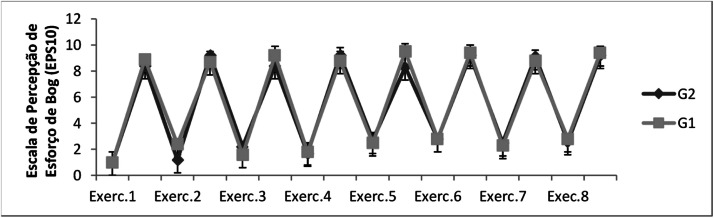
Evaluation of the Borg's Subjective Perceived Effort (SPE) of the sample of this study. Caption: Assessment of Borg's Perceived Exertion (SPE). Observation: results referring to the levels of subjective perception of effort in G1 and G2 before and after exercise. G1, Group that practiced water aerobics 1 × *a* week; G2, Group that practiced water aerobics 2 × *a* week. The symbol (*) indicates intragroup statistical differentiation. The significance level was *p* < 0.05.

The intensity of the interval exercise protocol in Borg Rating of Perceived Exertion is shown in Fig. 3. The Borg Rating of Perceived Exertion during the exercises behaved alternately, characterizing the interval method (*p* < 0.05). On G1 group, values increased significantly in Exercise 1 (1 ± 0.8 before; 8.9 ± 0.3 at the end); Exercise 2 (2.4 ± 0.2 before; 8.7 ± 0.5 at the end); Exercise 3 (1.6 ± 0.7 before; 9.2 ± 0.7 at the end); Exercise 4 (1.8 ± 0.5 before; 8.8 ± 0.7 at the end); Exercise 5 (2.5 ± 0.8 before; 9.5 ± 0.6 at the end); Exercise 6 (2.8 ± 0.3 before; 9.4 ± 0.6 at the end); Exercise 7 (2.3 ± 0.5 before; 8.8 ± 0.4 at the end); and Exercise 8 (2.8 ± 0.2 before; 9.4 ± 0.5 at the end). On G2 group, values also increased significantly in Exercise 1 (1 ± 0.7 before; 8.4 ± 0.4 at the end); Exercise 2 (1.2 ± 0.3 before; 9.2 ± 0.4 at the end); Exercise 3 (1.6 ± 0.6 before; 9.2 ± 0.8 at the end); Exercise 4 (1.7 ± 0.6 before; 9.2 ± 0.5 at the end); Exercise 5 (2.7 ± 0.8 before; 8.3 ± 0.4 at the end); Exercise 6 (2.8 ± 0.6 before; 9.2 ± 0.4 at the end); Exercise 7 (2.5 ± 0.5 before; 9.1 ± 0.2 at the end); and Exercise 8 (2.6 ± 0.6 before; 9.2 ± 0.4 at the end).

### Tests mental health

The mental health status of all participants is observed in the quality of sleep in G2, which reduced significantly (5.3 ± 1.7) when compared to pre-training (10.5 ± 1.1) scores. Regarding the size of the effect of physical exercise, the same group (G2) showed a 49 % increase in sleep quality. However, G1 did not present significant changes in post-training sleep quality (8.3 ± 1.2) compared to pre-training (10.8 ± 1.8) scores (*p* > 0.05).

Sleepiness Dormancy levels are given in Table 2. G2 significantly reduced (4.1 ± 1.8), when compared to the pre-training (8.3 ± 1.9) scores. Regarding the size of the effect of physical exercise in this group (G2), the authors observed a 50 % reduction in drowsiness symptoms. However, G1 did not change post-training sleepiness dormancy symptoms significantly (6.2 ± 1.5 scores) in comparison with pre-training (7.9 ± 1.1 scores) (*p* > 0.05).

**Table 2:** Characterization of the sample.

Variables	G1 (*n* = 21)	G2 (*n* = 35)	p-value
Age (years)	70.8 ± 5.5	67.2 ± 4.6	0.082
Body mass (kg)	76.9 ± 13.5	80.3 ± 12.6	0.091
Height (cm)	1.59 ± 10	1.52 ± 9	0.094
BMI (kg/m²)	30.9 ± 4.5	31.9 ± 3.4	0.082
Sex (M/F)	3/18	3/32	0.079
Morbidities			
Three pathologies	15 (71 %)	21 (60 %)	0.077
Two pathologies	6 (29 %)	14 (40 %)	0.062
Diabetics and hypertensive patients	3 (50 %)	4 (28 %)	0.095
Depressive and hypertensive patients	2 (33 %)	6 (42 %)	0.087
Depressive and diabetic patients	1 (17 %)	4 (28 %)	0.062
**Medical treatment**			
Losartan	13 (62 %)	22 (63 %)	0.082
Escitalopram	4 (19 %)	13 (37 %)	0.079
Bupropion	1 (5 %)	5 (14 %)	0.066
Fluoxetine	5 (24 %)	2 (6 %)	0.077
Amitriptyline	4 (19 %)	3 (9 %)	0.092
Clonazepam	7 (33 %)	5 (14 %)	0.094
Simvastatin	2 (10 %)	15 (43 %)	0.061
Enalapril	0 ( %)	8 (23 %)	0.000
Glibenclamida	9 (43 %)	29 (83 %)	0.082
Glifage	16 (76 %)	14 (40 %)	0.088

G1, Group that performed aqua gym 1 × *a* week; G2, Group that performed aqua gym 2 × *a* week. The significance level was *p* < 0.05.

Anxiety levels, as observed in Table 2, show that group G2 significantly reduced (8.0 ± 1.6), when compared to pre-training (14.6 ± 1.3) scores. Regarding the size of the effect of physical exercise in this group (G2), the authors observed a 45 % reduction in anxiety symptoms. However, G1 did not show significant score changes (11.6 ± 1.0) in post-training anxiety symptoms compared to pre-training (13.1 ± 1.6) (*p* > 0.05).

Table 2 also shows depression markers, where group G2 scores reduced significantly (6.6 ± 1.5), when compared to the pre-training (17.5 ± 1.9). Regarding the size of the effect of physical exercise in the same group (G2), the authors observed a 62 % reduction in depression symptoms. However, G1 did not significantly change post-training depression symptoms (13.4 ± 4.2 scores) compared to pre-training (15.1 ± 5.1) (*p* > 0.05).

## Discussion

The present study demonstrates that high-intensity interval aquatic training performed twice a week improves mental health parameters in older people with multimorbidity, while a frequency of once a week was insufficient to statistically improve any of the parameters analyzed. The present findings suggest that training frequency plays a crucial role in the benefits that exercise can provide. This may be due to the insufficient frequency of stimuli to promote the physiological and psychological adaptations observed with more frequent training protocols.

Two basic physiological variables used to control training intensity and prescription are heart rate.^22^^,^^23^ In Fig. 2, Fig. 3, the authors observe that both prescriptions are heart rate and Borg Rating of Perceived Exertion, behaved similarly (alternating ups and downs) during training sessions in both groups (G1 and G2), characterizing the high-intensity interval method. Coswig et al.^24^ demonstrated that a HIIT program (4 × 4 × 4 min) performed by older people similarly altered Heart Rate and Borg Rating of Perceived Exertion. Scherr et al.^25^ submitted men and women to physical training tests and found strong correlations between Borg Rating of Perceived Exertion and Heart Rate. It is a fact that the Heart Rate is directly related to Borg Rating of Perceived Exertion, increasing proportionally in the same direction during training.^23^^,^^26^ These elevations are related to the physiological activation of the sympathetic nervous system in response to training.^27^

Fluctuations in Heart Rate and Borg Rating of Perceived Exertion during high-intensity training indicate that the exercise protocol targets physiological zones promoting both physiological and psychological adaptations. These responses are critical for mental health, as elevated Heart Rate and Borg Rating of Perceived Exertion stimulate the release of neurotransmitters like endorphins, serotonin, and dopamine, which positively influence mood and psychological well-being.^28^ The activation of the sympathetic nervous system and subsequent hormonal responses help reduce stress, improve emotional regulation, and enhance mental resilience.^27^ Furthermore, the repetitive nature of high-intensity intervals may promote neuroplasticity, potentially supporting cognitive function and emotional stability in older people with multimorbidity. Although this remains a hypothesis, it warrants further investigation in future research.

Another line of investigation in the present manuscript is mental health, which can be positively or negatively impacted by the practice of exercise.^29^ These results, as given in Table 2, point to a 49 % improvement in sleep quality and a 50 % decrease in drowsiness symptoms in G2, the group that was submitted to a frequency of twice a week. Such results are in line with the findings of Delevatti et al.^30^ who concluded that interval water training for 12-weeks improves sleep quality, and Doyenart et al.^31^ who demonstrated that 8-weeks of interval water physical training (4 × 30 × 30 s) of moderate intensity improved aspects related to sleep and drowsiness in older people with diabetes. Jiménez-García et al.^32^ reported that the same population noticed that their sleep quality improved after undergoing a high-intensity interval exercise protocol (4 × 4 × 3 min) for 12 weeks. The physiological explanation for the present findings is that interval-based physical exercises promote circadian rhythm regulation, increase melatonin production, body temperature, and energy expenditure. Crucially, these exercises also enhance parasympathetic (vagal) activity, which plays a vital role in promoting relaxation and facilitating the transition to and maintenance of restorative sleep. Increased vagal tone is associated with reduced heart rate, muscle relaxation, and a shift towards the 'rest and digest' state, all of which are essential for improved sleep quality and reduced sleepiness.^33^, ^34^, ^35^, ^36^

However, anxiety disorders and depression are not rare in older people with multimorbidity.^37^ One study found that the risk of depressive disorder is two to three times higher in people with multimorbidity compared to those without multimorbidity.^38^ These findings (Table 3) point to an improvement of 45 % in anxiety after the interval training program twice a week (G2), and 62 % in depressive symptoms. Silva et al.^3^ found improvements in the mental health of depressed older people after 12-weeks of weekly low-intensity aquatic exercises (2 ×). In another study, Silva et al.^23^ found improvements in anxiety and depression levels in older people submitted to an aquatic exercise program (3 × 1 min × 30 s). The improvement in the symptoms of anxiety and depression could be explained by the social interaction and psychophysiological aspects provoked by the exercises performed in the water. Studies suggest that aquatic exercise, due to its low-impact nature and high cardiovascular stimulation, may be particularly effective in enhancing mental well-being in older people, providing both the physiological benefits of exercise and the therapeutic effects of water immersion.^6^^,^^23^^,^^39^

**Table 3:** Mental health parameters.

Parameters	G1	p-value	G2	p-value	%
PSQI (scores)					
**Pre**	10.8 ± 1.8		10.5 ± 1.1		
**Post**	8.3 ± 1.2	0.079	5.3 ± 1.7^a^	0.032	+49
ESS-BR (scores)					
**Pre**	7.9 ± 1.1		8.3 ± 1.9		
**Post**	6.2 ± 1.5	0.231	4.1 ± 1.8^a^	0.041	−50
Anxiety (BAI) (scores)					
**Pre**	13.1 ± 1.6		14.6 ± 1.3		
**Post**	11.6 ± 1.0	0.436	8.0 ± 1.6^a^	0.047	−45
Depression BDI (scores)					
**Pre**	15.1 ± 5.1		17.5 ± 1.9		
**Post**	13.4 ± 4.2	0.652	6.6 ± 1.5^a^	0.017	−62

G1, Group that performed aqua gym 1 × week; G2, Group that performed aqua gym 2 × week.

PSQI, Pittsburg Sleep Quality Index; ESS-BR, Epworth Sleepiness Scale for the Brazilian population; Anxiety (BAI), Beck Anxiety Scale; Depression (BDI), Beck Depression Inventory.

a Indicates an intragroup statistical difference. The significance level was *p* < 0.05.

These significant improvements in mental health parameters, including sleep quality, anxiety, and depressive symptoms, are particularly relevant for older adults with multimorbidity, as mental well-being is intrinsically linked to functional independence and engagement in daily life activities. Enhanced sleep quality provides more energy and reduces daytime fatigue, directly impacting the ability to perform routine tasks. Similarly, a reduction in anxiety and depressive symptoms can lead to increased motivation, improved cognitive function, and greater willingness to participate in social and physical activities.^28^ By fostering better mental health, high-intensity interval training, even at lower frequencies, can potentially break the vicious cycle of multimorbidity, mental health decline, and functional impairment, thereby facilitating the re-engagement of older adults in their activities of daily living and ultimately enhancing their overall quality of life.^40^ This direct translational impact underscores the clinical importance of interventions like aquatic HIIT for this vulnerable population.

This study has some limitations worth mentioning. Although the “1 × per week” group serves as a control for comparison purposes, future studies could consider additional strategies to better explore the effects of training frequency and intensity on multimorbid populations. Another limitation was the lack of stratification of participants based on specific multimorbidities, which could influence responses to exercise. Furthermore, greater rigor in detailing exercise execution and participant guidance would provide more actionable insights for exercise prescription. However, all outcome assessments were conducted in a blinded manner to minimize potential biases. Yet, the intervention itself was not blinded, as participants and instructors knew their training frequency. Finally, the authors did not weigh participants after the intervention, which limits the assessment of the relationship between weight loss and sleep quality in obese participants.

Despite these limitations, this study has several strengths. The structured high-intensity aquatic exercise program was well-tolerated by older people with multimorbidities, demonstrating their capacity to adapt effectively to such interventions. The adherence rate was high, and no adverse effects were reported, reinforcing the safety and feasibility of this exercise modality for this population.

As a scientific provocation, the authors suggest future research to explore: Which HIIT model delivers the most significant effects in older populations? Does the HIIT class model outperform continuous high-intensity exercise? How do older people with multimorbidities respond compared to those without such conditions? Addressing these questions could advance the understanding of the in-exercise prescription for this population.

## Conclusions

In older people with multimorbidity, High-Intensity Interval Training (HIIT) performed twice a week in an aquatic environment leads to improvements in mental health parameters. The present findings suggest that it is both safe and feasible for this population to engage in high-intensity exercises in the water. As a practical recommendation, the authors propose aqua aerobics classes twice a week, totaling 80 minutes, to enhance mental well-being. Additionally, the authors encourage further research to explore the impact of aquatic HIIT on cognitive function in this population.

## Ethics statement

This study was approved by the Research Ethics Committee of the local Institution and in the local Institutional and Brazilian Registry of Clinical Trials (ReBEC) RBR-9kqwd5f.

## Consent

The informed consent form was obtained from all the participants.

## Funding

No funding was received for this research.

## Authors’ contributions

Vitória Oliveira Silva da Silva: Formal analysis; investigation; writing-original draft; writing-review & editing.

Anand Thirupathi: Conceptualization; methodology; formal analysis; visualization; writing-review & editing.

Rafael Alex dos Santos Macedo: Investigation; resources.

Mauricio Fagundes Santos: Investigation; resources.

Ana Claudia de Oliveira Borba: Investigation; resources.

Yaodong Gu: Methodology; Formal analysis; data curation.

Karin Martins Gomes: Methodology; formal analysis; data curation.

Rodrigo Sudatti Delevatti: Formal analysis; writing-review & editing.

Paulo Cesar Lock Silveira: Conceptualization; funding acquisition; project administration; supervision; writing-review & editing.

Luciano Acordi da Silva: Conceptualization; funding acquisition; project administration; supervision; writing-review & editing.

## Declaration of competing interest

The authors declare no conflicts of interest.
